# A path to suicidal ideation through emotional intelligence: the mediating role of spiritual intelligence and resilience in borderline personality disorder

**DOI:** 10.3389/fpsyt.2026.1525688

**Published:** 2026-08-19

**Authors:** Faezeh Ghorbannejad, Noshirvan Khezri Moghaddam, Arvin Hedayati

**Affiliations:** 1 Department of Psychology, Shahid Bahonar University of Kerman, Kerman, Iran; 2 Department of Psychiatry, Shiraz University of Medical Sciences, Shiraz, Iran

**Keywords:** borderline personality disorder, emotional intelligence, resilience, spiritual intelligence, suicidal ideation

## Abstract

**Introduction:**

Low levels of emotional intelligence may constitute a vulnerability factor associated with suicidal ideation. However, the mechanisms underlying this relationship require further investigation. This study explored how spiritual intelligence and resilience mediate the relationship between emotional intelligence and suicidal ideation in individuals with borderline personality disorder.

**Methods:**

To achieve this goal, 161 participants (18–50 years old) completed the questionnaire: The Siberia Schering’s Emotional Intelligence, The King Spiritual Intelligence Questionnaire (SISRI24), The Connor-Davidson Resilience Scale (CD-RISK), and The Beck Scale for Suicide Ideation (BSSI) measuring the study variables. We conducted this research using SPSS 26 and Macro v4.3.

**Results:**

Results revealed that there was a significant and negative relationship between emotional intelligence and suicidal ideation. Furthermore, it was found that spiritual intelligence and resilience mediate the relationship between emotional intelligence and suicidal ideation. However, there was no significant association between emotional intelligence and suicidal ideation via spiritual intelligence.

**Discussion:**

The findings of this study align well with most of our hypotheses, highlighting the role of emotional intelligence in relation to suicidal ideation and emphasizing the importance of spiritual intelligence and resilience as mediating factors.

## 1 Introduction

Suicidal behaviors pose a significant public health challenge worldwide, inflicting severe personal, social, and economic damage on society (1). According to the National Institute of Mental Health (NIMH), suicide is defined as death caused by self-directed injurious behavior with intent to die as a result of the behavior (2). Suicidal behavior is categorized into suicidal ideation, attempted suicide, and completed suicide (3). According to the latest World Health Organization (WHO) report, approximately 727, 000 people died by suicide worldwide in 2021, accounting for more than one in every 100 deaths (1.1%) globally (4). Findings from Lim et al., presented in a meta-analysis covering global life expectancy and the twelve-month prevalence of suicidal behaviors between 1989 and 2018, indicate a prevalence rate of 6% for suicide attempts, 9.9% for suicide plans, and 22% for suicidal thoughts and ideation (5). It is important to acknowledge that suicide statistics are often underestimated due to various moral, cultural, and religious factors (6).

Suicidal behavior is a complex, multifaceted issue influenced by various factors. Early research into the risk factors for suicidal actions centered on elements such as genetic defects and disorders, adverse childhood experiences encompass a range of traumas, including sexual, physical, or verbal abuse, neglect, bullying, and other challenging life events. These may also involve the loss of meaningful relationships or struggles encountered in school or the workplace (7, 8).

Beyond these, personality traits like impulsivity, low self-esteem, perfectionism, and hopelessness, as well as psychological, social, cultural factors, physical and family issues, environmental conditions, and various stressors faced by individuals, play a role in suicidal behavior. A history of attempted suicide or self-injurious behavior without the intent to die can also contribute to the risk (9–12). Numerous studies highlight that one of the most significant predictors of suicidal ideation is having personality disorders—particularly borderline personality disorder (BPD). This disorder includes suicidal or self-injurious behaviors as diagnostic criteria (13–16). Borderline personality disorder is characterized by instability in many life areas, including interpersonal relationships, emotions, and behavior. It often leads to severe functional impairment, substantial mental and physical disability, and an increased likelihood of self-destructive and health-damaging behaviors (17).

The lifetime prevalence of borderline personality disorder is estimated to range from about 0.5% to 6% in the general population, with a recent meta-analysis estimating a global prevalence of 1.8%. However, the condition appears more frequently among hospitalized patients, with prevalence rates around 10%–20% (18). Emotional dysregulation is one of the core clinical characteristics of borderline personality disorder (BPD), contributing to heightened emotional sensitivity and difficulties in regulating emotional responses (17). Consistent with this characteristic, individuals with BPD have been shown to exhibit significant deficits across multiple domains of emotional intelligence (EI), particularly in emotion regulation (19). Given that EI reflects the ability to perceive, understand, and regulate emotions, it may represent a particularly relevant construct for investigating suicidal ideation in this population. Emotional intelligence also involves effectively utilizing emotional information to facilitate adaptive self-management and interpersonal functioning (20). According to Goleman’s model, emotional intelligence comprises self-awareness, self-regulation, motivation, empathy, and social skills. Deficits across these EI domains have been associated with maladaptive coping, heightened emotional reactivity, and suicidal ideation (21). Prior studies suggest that higher emotional intelligence is associated with lower levels of suicidal behaviors, whereas lower emotional intelligence has been associated with greater suicidal risk, including among individuals with BPD (22–24). These findings suggest that higher EI may promote psychological adjustment by enhancing coping with stressful experiences and facilitating the use of more adaptive coping strategies, which have been associated with lower suicidal risk (24–27).

Among the core diagnostic features of borderline personality disorder (BPD), chronic feelings of emptiness are recognized as a core and clinically significant feature of the disorder (17). Despite its clinical importance, chronic emptiness has received considerably less empirical attention than other core features of BPD, and its underlying psychological mechanisms remain poorly understood (28). Nevertheless, previous studies have associated chronic emptiness with hopelessness, interpersonal dysfunction, self-harming behaviors, and suicidal behaviors, highlighting the need to investigate psychological factors that may contribute to this clinically significant symptom (28, 29).

Given that experiences of emptiness are often characterized by a lack of meaning and hope, meaning in life and spirituality have increasingly been recognized as important psychological resources that may facilitate psychological recovery and reduce suicidal vulnerability among individuals with mental disorders (30–33). Nevertheless, despite growing recognition of their potential clinical value, the role of spirituality and related constructs in the psychological functioning of individuals with borderline personality disorder remains insufficiently investigated. In this context, spiritual intelligence, which reflects the capacity to apply spiritual resources to understand life experiences, construct personal meaning, and cope adaptively with adversity, may represent a relevant psychological construct worthy of further investigation in individuals with borderline personality disorder (34–36).

Spiritual intelligence is more than merely combining intellect with spirituality; it encompasses a set of higher-order cognitive abilities and adaptive personal resources that facilitate meaning-making, existential understanding, and effective coping with life challenges, thereby promoting psychological functioning and well-being. Previous studies have shown that higher levels of spiritual intelligence are associated with better mental health outcomes and lower suicidal ideation, suggesting its potential role as a protective psychological resource (37, 38). According to King, spiritual intelligence comprises four core dimensions: critical existential thinking, producing personal meaning, transcendental awareness, and conscious state expansion (39). Finally, emerging evidence suggests that spiritual intelligence may interact with emotional intelligence to enhance adaptive coping capacities and reduce vulnerability to suicidal ideation by facilitating meaning-based coping and more effective emotional regulation when individuals encounter stressful life events (40, 41).

Research indicates that resilience, alongside emotional intelligence, plays a crucial role in preventing suicidal behaviors by supporting mental health (42). Resilience reflects an individual’s ability to sustain mental and physical performance when confronted with difficult life situations. It enables one to tackle challenges and problems effectively, using healthier and safer methods (43). Initially resilience was considered only as an individual trait, but now it is understood as resulting from the intricate interplay between personal and environmental factors. Consequently, resilience can be defined as the ability to harness available environmental resources to manage and overcome difficulties (44). Emotional intelligence and resilience are interrelated and mutually supportive concepts. Studies suggest that individuals with high emotional intelligence often exhibit greater resilience when facing life’s challenges. These individuals tend to understand and manage their emotions effectively, which empowers them to adapt and address problems more capably (45).

Individuals who are adept at understanding and being aware of their emotions tend to manage their reactions more effectively in stressful situations and communicate better with others. This deep emotional awareness fosters greater empathy and enhances social relationships, an essential component in bolstering resilience (46). Furthermore, those with high emotional intelligence often develop superior emotion regulation skills. Rather than succumbing to stress and challenges, they are able to respond more positively by employing effective coping strategies. This emotional management contributes to increased resilience and balance in life (47). Conversely, previous studies have reported that resilience is positively associated with emotional intelligence, suggesting that these constructs may reinforce one another (48). These individuals also possess a high level of internal motivation and satisfaction with life, which, along with social support, plays a crucial role in linking emotional intelligence and resilience (49). Additionally, research over recent decades indicates that higher levels of resilience are associated with reduced suicidal behaviors (50–52).

Most studies have focused on the role of spirituality rather than spiritual intelligence as a broader and more inclusive concept in the context of suicidal behaviors. Additionally, there has been limited research on resilience and spiritual intelligence as mediating factors in the link between emotional intelligence and suicidal tendencies. To address this gap, the present study seeks to explore the indirect effects of spiritual intelligence and resilience on the relationship between emotional intelligence and suicidal tendencies in adults with borderline personality disorder. It also aims to offer a theoretical framework for preventing and intervening in suicidal behaviors.

**Figure 1:**
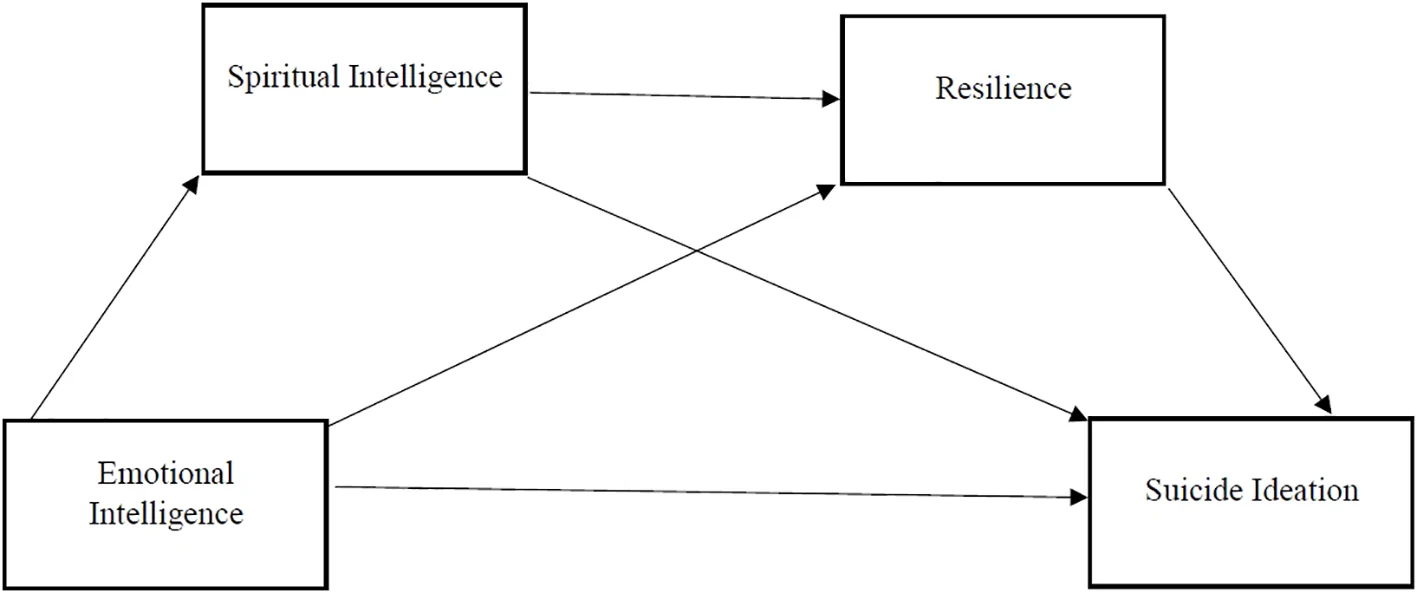
The hypothetical structure model.

The hypotheses of this research are:

1. Emotional intelligence has a direct relationship with suicidal ideation.
2. Emotional intelligence is indirectly linked to suicidal ideation via spiritual intelligence.
3. Emotional intelligence is indirectly associated with suicidal ideation through resilience.
4. Emotional intelligence is indirectly associated with suicidal ideation through both spiritual intelligence and resilience (see **Figure 1**).

## 2 Materials and methods

### 2.1 Research design and participants

This cross-sectional study was conducted between April and October 2023. After a brief screening to confirm eligibility, participants completed paper-based self-report questionnaires in person. In this study 161 patients with borderline personality disorder (96 men and 65 women) were selected from among those who referred to three neuropsychological Hospitals in Shiraz, Iran, using available sampling method, in the age range of 18 to 50 year (Mean age = 29.80 ± 0.6). The research inclusion criteria included providing informed consent, willingness to participate, and have a primary diagnosis of borderline personality disorder confirmed by a psychiatrist as the main diagnosis. Exclusion criteria included participant dissatisfaction, intellectual disability, not completing the questionnaires accurately, and lack of full consciousness to answer questions due to drug influence or concurrent psychotic disorders. The socio-demographic characteristics of the participants are presented in **Table 1**.

**Table 1:** Socio-demographic characteristics of samples (N = 161).

Variables	Categories	Frequency (%)
Gender	Female	65(40.4)
	Male	96(59.6)
Marital Status	Single	114(70.8)
	Married	36(22.4)
	Divorced	11(6.8)
Age Group (year)	18-30	95(59)
	31-40	50(31.1)
	41-50	16(9.9)
Education Level	Elementary	17(10.6)
	Under diploma	35(21.7)
	Diploma	81(50.3)
	Bachelor	22(13.7)
	Masters and above	6(3.7)

### 2.2 Research implementation method

In alignment with the Declaration of Helsinki, after obtaining ethical code from the Shiraz University of Medical Sciences ethics committee (IR.SUMS.REC. 1402. 026), alongside other requisite permissions, we reviewed the files of patients diagnosed with borderline personality disorder and conducted preliminary interviews with them. The patients were informed about the research purpose and assured of the confidentiality of their responses. After obtaining their informed consent, the questionnaires were distributed to the participants. They had the option to withdraw from the study at any time of the research.

### 2.3 Measurement tools

Demographic Information Questionnaire: This questionnaire included details such as gender, age, marital status and education level.

Emotional Intelligence Questionnaire: The *Siberia* Schering’s Emotional Intelligence Questionnaire is based on Goleman’s (1995) model of emotional intelligence, which evaluates emotional intelligence through five components: self-awareness, self-regulation, self-motivation, social awareness (empathy), and social skills. It uses a five-point Likert scale (53). The Iranian version of this questionnaire contains 33 questions, with the overall emotional intelligence score ranging from 33 to 165. A higher score in each subscale indicates its significance in an individual’s emotional intelligence. This version was assessed by Mansouri, with a Cronbach’s alpha of 0.85, aligning with results from other evaluations (54–56).

The Spiritual Intelligence Questionnaire: The King Spiritual Intelligence Questionnaire, developed in 2008, consists of 24 items. This questionnaire evaluates four main dimensions of spiritual intelligence: critical existential thinking, producing personal meaning, transcendent awareness, and conscious state expansion, along with an overall score of spiritual intelligence, using a five-point Likert scale (57). The total spiritual intelligence score ranges from 0 to 96. King assessed the reliability of this test using Cronbach’s alpha coefficient, achieving a score of 0.92 (58).

The Connor-Davidson Resilience Scale is a questionnaire consisting of 25 items rated on a five-point Likert scale. Developed by Connor and Davidson in 2003, its purpose is to assess the level of resilience in individuals. The authors validated its reliability through factor analysis and both convergent and divergent validity. They reported a Cronbach’s alpha of 0.89 and a one-month retest reliability of 0.87 (59). This scale measures resilience across five components: personal competence/high standards, positive acceptance of change and secure relationships, spiritual influences, control, and trust in one’s instincts/tolerance of negative emotions, while also providing an overall resilience score. Scores range from 0 to 100, where higher scores indicate greater resilience (60).

Suicidal Ideation: The Beck Scale for Suicide Ideation (BSSI) in its Iranian version is a 19-question self-assessment tool set on a three-point scale, initially developed by Aaron Beck in 1979 (61). This widely used questionnaire aims to measure the intensity of attitudes, behaviors, and planned intentions related to suicide attempts over the past week. It evaluates aspects such as the ability to control suicidal thoughts, the readiness to attempt suicide, as well as the duration and frequency of these thoughts (62). The Cronbach’s alpha reliability of the Iranian version was reported by Khosravi to be 0.90 (19).

### 2.4 Data analysis

After collecting questionnaires from participants, all analyses were conducted using SPSS version 26. Due to the use of self-report tools, the initial step involved examining common method variance that can lead to incorrect correlations. To address this issue, the Harman’s single-factor test was conducted based on the suggestion of Chang and colleagues (63). Then we assessed descriptive analyses to explore missing data, normality, multicollinearity. To investigate the bivariate relationships among the main variables and their sub-scales, Pearson correlation coefficients were calculated. Furthermore, to identify the unique predictive power of each sub-scale, a multiple linear regression analysis was conducted with all sub-scales entered simultaneously as predictors of suicidal ideation. Finally, to test the hypothesized serial mediating effects, we employed Hayes’ Process Macro for SPSS (Model 6) with 10, 000 bootstrap samples and bias-corrected 95% confidence intervals (64).”

## 3 Results

### 3.1 Initial analyses

No data was missing. According to studies, if the total variance extracted by one factor exceeds 50%, common method variance is present in the research (65). In this study, the average variance extracted for the surveyed items was 16.81%, which was below the recommended threshold of 50%. Thus, it can be concluded that common method bias was not evident. We evaluated the normality of the data by examining the skewness and kurtosis values. The absolute values for skewness and kurtosis all remained below ±2, so the data distribution was considered normal (66).

### 3.2 Correlation analysis

**Table 2** presents the descriptive statistics of all main study variables, along with their Pearson correlation coefficients with suicidal ideation. As shown, emotional intelligence (r = -0.555, p < 0.01), spiritual intelligence (r = -0.257, p < 0.01), and resilience (r = -0.501, p < 0.01) were significantly and negatively correlated with suicidal ideation. Additionally, significant positive associations were observed between emotional intelligence with spiritual intelligence (r = 0.503, p < 0.01) and resilience (r = 0.595, p < 0.01). **Table 3** reports the Pearson correlations between each sub-scale of emotional intelligence, spiritual intelligence, and resilience, and suicidal ideation. The results indicate that most sub-scales were significantly correlated with suicidal ideation (p<.01), whereas two sub-scales of spiritual intelligence (transcendent awareness and critical existential thinking) did not show a significant direct association. Given these bivariate associations, a multiple linear regression analysis was subsequently conducted to determine which specific sub-scales retain significant predictive power for suicidal ideation when controlling for the variance shared among other predictors.

**Table 2:** Descriptive statistics and correlation of the main study variables.

Variables	Mean ± SD	Skewness	Kurtosis	1	2	3	4
1- Emotional Intelligence	106.23 ± 18.23	- 0.063	- 0.980	1			
2- Spiritual Intelligence	57.97 ± 11.69	- 0.119	- 0.105	0.503^**^	1		
3- Resilience	56.66 ± 16.28	- 0.420	- 0. 293	0.595^**^	0.555^**^	1	
4- Suicide Ideation	8.74± 10.75	0. 925	- 0.505	-0.555^**^	-0.257^**^	-0.501^**^	1

** p < 0.01.

**Table 3:** Correlations between sub-scales independent variables and suicide ideation.

Variables	Sub-scales	Suicide ideation
Emotional Intelligence	self-awareness	-.499^**^
	self-regulation	-.517^**^
	self-motivation	-.404^**^
	social awareness	-.445^**^
	social skills	-.305^**^
Resilience	personal competence	-.471^**^
	positive acceptance of change	-.438^**^
	spiritual influences	-.328^**^
	control	-.444^**^
	tolerance of negative emotions	-.320^**^
Spiritual Intelligence	critical existential thinking	-.026
	producing personal meaning	-.403^**^
	transcendent awareness	-.086
	conscious state expansion	-.258^**^

**Correlation is significant at the 0.01 level (2-tailed).

### 3.3 Multiple regression analysis

To identify the unique predictive power of each sub-scale, a multiple linear regression analysis was conducted with all 14 sub-scales entered simultaneously as predictors of suicidal ideation. The overall model was statistically significant, F (14, 146) =7.308, p<.001, and explained approximately 36% of the variance in suicidal ideation (*R*^2^ = .412, Adj. *R*^2^ = .356 As presented in **Table 4**, five sub-scales emerged as significant unique predictors of suicidal ideation at the p <.05 level. These included two Emotional Intelligence sub-scales, self-awareness (β = −.263) and self-regulation (β = −.327); one Spiritual Intelligence sub-scale, producing personal meaning (β = −.457); and two Resilience sub-scales, positive acceptance (β = −.193) and personal competence (β = −.255). All five predictors showed significant negative relationships with suicidal ideation.

**Table 4:** Summary of multiple linear regression analysis predicting suicidality from sub-scales scores.

Variables		Unstandardized coefficient B	Standardized coefficient Beta	t	Sig.	VIF
Emotional Intelligence	self-awareness	-.624	-.263	-2.741	.007	2.411
	self-motivation	-.157	-.077	-.889	.375	1.706
	self-regulation	-.612	-.327	-3.414	.023	2.503
	social awareness	-.377	-.136	-1.482	.140	1.909
	social skills	-.091	-.029	-.365	.715	1.429
Resilience	personal competence	-.441	-.255	-2.393	.018	2.449
	positive acceptance	-.515	-.193	-2.014	.046	1.986
	spiritual influences	-.535	-.111	-1.374	.171	1.398
	control	-.579	-.156	-1.595	.113	2.067
	tolerance of negative emotions	.215	.091	.947	.345	1.986
Spiritual Intelligence	critical existential thinking	.379	.157	1.754	.081	1.567
	producing personal meaning	-1.277	-.457	-4.979	.000	1.658
	transcendent awareness	.357	.146	1.464	.145	1.955
	conscious state expansion	-.456	-.149	-1.506	.134	1.914

### 3.4 Mediation analysis

**Figure 2:**
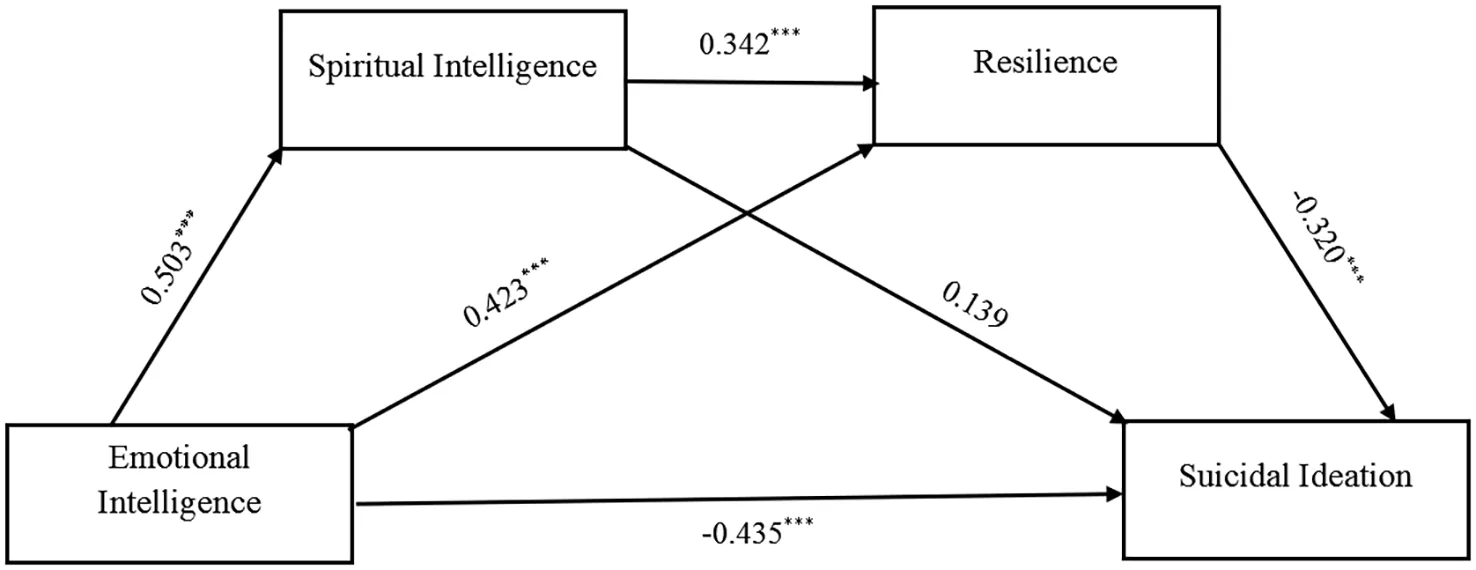
Mechanism of emotional intelligence on suicidal ideation. ***p < 0.001.

To further investigate the underlying mechanisms through which emotional intelligence influences suicidal ideation, a serial mediation model was tested using Hayes’ Process Macro (Model 6). This study was performed to examine the mediating effects of spiritual intelligence and resilience on the relationship between emotional intelligence and suicidal ideation among borderline personality disorder patients (**Table 5**; **Figure 2**).

**Table 5:** Serial mediating effect of spiritual intelligence and resilience on emotional intelligence and suicidal ideation.

Path	B	SE	t	LLCI	ULCI
Emotional Intelligence → Spiritual intelligence	0.503	0.044	7.330^***^	0.236	0.409
Emotional Intelligence → Resilience	0.423	0.061	6.153^***^	0.257	0.500
Spiritual intelligence → Resilience	0.342	0.096	4.973^***^	0.287	0.665
Emotional Intelligence → Suicide Ideation	-0.435	0.048	- 5.311^***^	-0.352	-0.161
Spiritual intelligence → Suicide ideation	0.139	0.073	1.763	-0.015	0.272
ResilienceSuicide ideation	-0.320	0.056	- 3.764^***^	-0.322	-0.101

*** p < 0.001.

As presented in **Table 5** the results indicate that emotional intelligence significantly and positively influenced spiritual intelligence (β = 0.503, p < 0.001) and resilience (β = 0.423, p < 0.001), while negatively predicting suicidal ideation (β = −0.435, p < 0.001). Moreover, resilience negatively predicted suicidal ideation (β = -0.320, p < 0.001). Although the effect of spiritual intelligence on both resilience and suicidal ideation was not significant. Using 95% bootstrap confidence intervals from 10, 000 bootstrap replications, the total serial mediating effect of this study was - 0.120 which was significant duo to no 0 between LLCI and ULCI (- 0.242 - -0.013). As presented in **Table 6** verifying other mediation paths revealed that the path from emotional intelligence to suicidal ideation via resilience was significant (B = - 0.135; - 0.233 to -0.057), while the path from emotional intelligence to suicidal ideation via spiritual intelligence was not significant due to 0 between LLCI and ULCI. However indirect effect through both spiritual intelligence and resilience was significant because of not encompassing zero (B = - 0.055; - 0.112 to -0.019).

**Table 6:** Indirect, direct and total effects of mediation model.

Path	Effect	SE	LLCI	ULCI
EI → SI → Suicide Ideation	0.070	0.042	-0.007	0.158
EI → Res → Suicide Ideation	0.135	0.046	-0.233	-0.057
EI → SI → Res → Suicide Ideation	0.055	0.024	-0.112	-0.019
Total Indirect Effect	0.120	0.058	-0.242	-0.013
Direct Effect: SI → Suicide Ideation	0.256	0.048	-0.352	-0.161
Total effect	0.327	0.039	-0.404	-0.251

EI, Emotional Intelligence, SI, Spiritual intelligence, Res, Resilience.

## 4 Discussion

This study investigated the relationships among emotional intelligence, spiritual intelligence, resilience, and suicidal ideation in patients with borderline personality disorder (BPD). Furthermore, it examined the serial mediating effects through spiritual intelligence and resilience in the pathway from emotional intelligence to suicidal ideation. Preliminary analyses revealed significant negative correlations between emotional intelligence, spiritual intelligence, and resilience with suicidal ideation, providing the basis for testing the hypothesized mediation models.

Multiple Regression: One of the key findings of this study was the identification of five specific sub-scales that uniquely predicted suicidal ideation among patients with borderline personality disorder (BPD). Contrary to our expectation that emotional intelligence dimensions, particularly those related to emotion regulation, would emerge as the strongest predictors, producing personal meaning from spiritual intelligence emerged as the strongest negative predictor of suicidal ideation, showing a stronger association than the other significant emotional intelligence and resilience subscales identified in the present study. This finding suggests that meaning-making may be particularly relevant in understanding suicidal ideation among individuals with BPD. In BPD, chronic feelings of emptiness and a sense of inner void have been associated with suicidal ideation (28). Accordingly, the present finding may reflect the importance of meaning-making as a psychological resource. Previous theoretical and empirical work suggests that constructing personal meaning may facilitate adaptive coping with psychological distress and is associated with lower suicidal ideation (30–32).

Following meaning-making, self-regulation and self-awareness from emotional intelligence, along with personal competence and positive acceptance from resilience, were also identified as significant negative predictors of suicidal ideation. These findings suggest that, in addition to meaning-making, emotional and resilience-related capacities may also be associated with lower suicidal ideation among individuals with BPD. The significant role of self-regulation observed in the present study is consistent with previous evidence identifying emotion dysregulation as a core clinical feature of BPD (22). Self-regulation, which refers to the ability to effectively manage emotional responses, has been associated with more adaptive coping and better psychological adjustment in previous research (19, 22, 25). Likewise, self-awareness has been associated with the recognition and understanding of one’s emotional experiences and with more adaptive emotional functioning in previous research (23, 25). Although the present study did not examine the underlying mechanisms linking these constructs to suicidal ideation, these findings are consistent with previous literature suggesting that emotional awareness and self-regulation may represent potential protective psychological resources in individuals with BPD (19, 22, 67).

On the other hand, resilience components, specifically personal competence and positive acceptance, were also identified as significant negative predictors of suicidal ideation. These findings suggest that resilience-related capacities may also contribute to lower suicidal ideation among individuals with BPD. Personal competence refers to individuals’ confidence in their ability to cope effectively with life challenges (59). One possible explanation for the present finding is that greater personal competence may strengthen individuals’ perceived ability to cope with psychological crises, thereby fostering hope and reducing feelings of helplessness. This interpretation is consistent with previous studies highlighting resilience as a protective factor against suicidal ideation and suicidal behavior (43, 51, 52, 68). Positive acceptance, which reflects the ability to accept oneself and life experiences in an adaptive manner (59), may also facilitate more adaptive psychological adjustment. Previous studies have similarly suggested that resilience-related capacities are associated with better psychological well-being and lower suicidal risk (49, 50). Although the present study did not directly examine the mechanisms underlying these associations, the current findings are consistent with previous literature suggesting that resilience may represent an important protective psychological resource against suicidal ideation.

Overall, the present findings suggest that multiple psychological domains may be differentially associated with suicidal ideation among individuals with borderline personality disorder (BPD), with meaning-making, emotional functioning, and resilience-related capacities emerging as particularly relevant. Among the variables examined, producing personal meaning showed the strongest unique association with suicidal ideation, followed by self-regulation, self-awareness, personal competence, and positive acceptance. Notably, the remaining nine sub-scales did not demonstrate significant unique predictive power after controlling for the other predictors, suggesting that their associations with suicidal ideation were largely shared with these five dimensions. These findings highlight the potential value of examining specific dimensions of emotional intelligence, spiritual intelligence, and resilience, rather than relying solely on global construct scores, when investigating suicidal ideation among individuals with BPD (23, 39).

Hypothesis 1: The results of the present study confirmed hypothesis 1 and were consistent with previous research (9, 19, 67, 69–71), suggesting that lower levels of emotional intelligence may be associated with increased suicidal ideation, particularly among individuals with borderline personality disorder. Emotional intelligence, defined as the capacity to perceive, understand, and regulate one’s own and others’ emotions, plays a pivotal role in shaping the quality of social interactions and adaptive functioning. Specifically, higher levels of emotional intelligence facilitate the recognition of appropriate emotional responses to daily stressors, broaden cognitive insight, and foster a more positive outlook on life challenges (69). One possible explanation for these findings is that individuals with higher emotional intelligence may be better able to recognize, understand, and regulate their emotional experiences, thereby facilitating more adaptive responses to psychological distress. Previous studies have similarly suggested that emotional intelligence is associated with emotional awareness, adaptive coping, and lower suicide risk (67, 69, 71).

Conversely, individuals with lower emotional intelligence often struggle with emotional awareness, problem-solving, and empathy, leading to a limited repertoire of coping strategies and reduced social support in managing crises (71). In addition, the present findings suggest that emotional intelligence may also be positively associated with spiritual intelligence. Individuals with higher emotional intelligence may be better able to engage in meaning-making and adaptive coping processes, which have been proposed as important protective psychological resources against suicidal ideation in previous studies (24, 57).

Hypothesis 2: Contrary to Hypothesis 2 and most previous studies (41, 72, 73), which have generally reported an inverse relationship between spiritual intelligence and suicidal ideation, the present findings indicated that neither the direct association between spiritual intelligence and suicidal ideation nor the indirect pathway through spiritual intelligence was statistically significant. This unexpected finding suggests that the overall construct of spiritual intelligence may not function as a direct protective factor against suicidal ideation among individuals with BPD.

Importantly, however, this finding should be interpreted alongside the regression results. Although the overall spiritual intelligence score was not a significant predictor, the producing personal meaning subscale emerged as the strongest negative predictor of suicidal ideation in the present study. Taken together, these findings suggest that the protective role of spiritual intelligence may not be uniformly distributed across all of its dimensions. Rather, specific components related to meaning-making may be more relevant to suicidal ideation than the global construct itself. This interpretation is consistent with previous theoretical and empirical work emphasizing that the ability to construct meaning from life experiences represents one of the central psychological resources underlying resilience and protection against suicide (30–32).

One possible explanation for the discrepancy between the non-significant global effect and the significant role of producing personal meaning is that the overall spiritual intelligence score may combine several distinct dimensions with different psychological functions. While these dimensions collectively contribute to the construct of spiritual intelligence, they may not all be equally relevant to suicidal ideation. In contrast, the capacity to derive personal meaning from adverse experiences may represent the specific aspect of spiritual intelligence most directly associated with protection against suicidal thoughts in individuals with BPD. This interpretation is also compatible with King’s conceptualization of spiritual intelligence, which proposes that its dimensions represent related but distinct higher-order cognitive capacities rather than interchangeable components (31, 74).

Given that chronic feelings of emptiness and identity disturbance are among the core clinical characteristics of BPD (17), the ability to construct personal meaning may be particularly important in this population. Previous research has similarly suggested that meaning-making is associated with better psychological adjustment, greater resilience, and lower suicidal ideation (30–32). Although the present study did not directly examine these underlying mechanisms, the current findings are consistent with this body of literature and suggest that future research should investigate whether meaning-making represents the principal pathway through which spiritual resources contribute to reduced suicide risk in BPD.

Hypothesis 3: The findings bolster Hypothesis 3 and the past literature that there is a significant negative association between suicidal ideation and resilience (50–52, 68). Resilience is the process and outcome of successfully adapting to difficult or challenging life experiences, especially through psychological, emotional, and behavioral flexibility and adaptation to external and internal demands (50). Importantly, resilience acted as a significant mediating pathway through which emotional intelligence was associated with lower suicidal ideation, thereby linking emotional competence with better psychological adaptation.

The present findings further suggest that this mediating effect may be particularly reflected in the resilience dimensions of personal competence and positive acceptance, both of which emerged as significant negative predictors of suicidal ideation in the regression analysis. Rather than the global construct of resilience alone, these specific dimensions appear to be more closely associated with suicidal ideation in individuals with BPD. Personal competence, which reflects confidence in one’s ability to cope successfully with adversity, has previously been associated with greater psychological adjustment, more effective coping, and lower vulnerability to psychological distress (51, 52, 59). Likewise, positive acceptance has been linked to greater psychological flexibility, self-acceptance, and adaptive adjustment following stressful experiences (59, 60).

Although the present study did not directly examine the psychological mechanisms underlying these relationships, the current findings are consistent with previous literature suggesting that individuals with greater personal competence and positive acceptance may be better equipped to cope with emotional distress and life challenges, thereby experiencing lower levels of suicidal ideation (49, 50, 68). Taken together, these findings suggest that resilience may represent an important pathway through which emotional intelligence is associated with suicidal ideation. Specifically, the dimensions of personal competence and positive acceptance appear to play a particularly relevant role, highlighting the importance of strengthening these resilience-related capacities when designing psychological interventions for individuals with BPD.

Hypothesis 4: Finally, the research supports Hypothesis 4, indicating that emotional intelligence is associated with suicidal ideation through spiritual intelligence and resilience as mediators. The serial mediation model identified in the present study suggests that emotional intelligence may be associated with suicidal ideation through a sequence involving spiritual intelligence and resilience. These findings are consistent with the conceptual framework proposed in the present study, in which emotional intelligence represents an important psychological resource associated with other adaptive psychological capacities, including resilience and spiritual intelligence (23, 25). As discussed in Hypothesis 2, the protective association of spiritual intelligence may be particularly reflected in the dimension of producing personal meaning (30, 32), whereas resilience appears to represent an important pathway linking emotional intelligence with lower suicidal ideation (19, 25). Taken together, these findings suggest that emotional intelligence may be associated with lower suicidal ideation both directly and indirectly through resilience and the meaning-making dimension of spiritual intelligence, supporting the proposed serial mediation model.

This finding aligns with numerous studies that have examined the role of these factors in predicting suicidal ideation (13, 19, 75–77). However, these findings should be interpreted within the context of a cross-sectional mediation model and therefore do not establish causal relationships among the study variables (64). It should also be noted that this model explains only a portion of the variance in suicidal ideation. Many other vulnerability and risk factors may contribute to suicidal ideation, including adverse family environments, lack of social support, childhood physical and sexual abuse, genetic vulnerability, and co-occurring psychiatric disorders such as depression (8, 10, 78). Therefore, the present findings should be interpreted as highlighting several potentially important psychological resources associated with suicidal ideation rather than providing a comprehensive explanation of suicide risk in individuals with BPD.

From a clinical perspective, these findings suggest that mental health interventions for BPD patients should not only focus on symptom management or pharmacotherapy but also incorporate targeted training to enhance emotional intelligence, foster meaningful spiritual engagement, and build specific dimensions of resilience. Given that producing personal meaning emerged as the strongest predictor in our regression analysis, therapeutic approaches should move beyond general spiritual education to actively promote meaning-making skills. This implies that therapists may help patients utilize their emotional intelligence to regulate intense negative emotions and then channel this regulated energy into constructing personal purpose and existential clarity.

Furthermore, since personal competence and positive acceptance were identified as key resilience components, clinical programs should focus on boosting patients’ sense of efficacy and reducing self-blame. This could be achieved through counseling strategies, emotion regulation training, and mindfulness-based interventions that encourage self-compassion and the acceptance of one’s strengths and weaknesses. Such an integrated approach may strengthen resilience and support psychological adaptation by enhancement of personal meaning, self-acceptance, and personal competence. Additionally, educational policies could integrate these psychological skills (emotional intelligence, meaning-making, and resilience) at earlier stages of development to promote psychological well-being and potentially reduce suicidal tendencies among at-risk populations, especially adolescents (38, 79).

In summary, the present study supports the proposed model that emotional intelligence is associated with suicidal ideation in individuals with borderline personality disorder through distinct psychological pathways involving resilience and spiritual intelligence. The findings suggest that emotional intelligence may be associated with lower suicidal ideation both directly and indirectly by strengthening resilience—particularly the dimensions of personal competence and positive acceptance—and through the meaning-making dimension of spiritual intelligence. Importantly, while global spiritual intelligence did not demonstrate a significant direct association with suicidal ideation, producing personal meaning emerged as the strongest negative predictor, highlighting the importance of meaning-making rather than spiritual awareness alone. Taken together, these findings contribute to a more comprehensive understanding of suicidal ideation in BPD by emphasizing the potential roles of emotional functioning, resilience, and meaning-making as interconnected psychological resources. From a clinical perspective, these findings suggest that psychological interventions targeting emotional intelligence, resilience, and meaning-making may complement pharmacological treatment and contribute to suicide prevention efforts in individuals with BPD. Future research using longitudinal and experimental designs is needed to clarify the causal mechanisms underlying these relationships.

### 4.1 Limitation

This study examined the correlational relationships among factors associated with suicidal ideation among patients with borderline personality disorder (BPD). However, several limitations should be acknowledged.

First, all data were gathered through self-reported questionnaires. Conducting clinical interviews could provide a more comprehensive understanding of patients’ psychological functioning and complement self-report assessments. Second, although the present study examined the predictive role of individual subscales in the multiple regression analysis, the serial mediation model was tested using the global constructs of emotional intelligence, spiritual intelligence, and resilience. Future studies could examine mediation models at the subscale level to determine whether specific dimensions operate through distinct psychological pathways in relation to suicidal ideation. Third, all participants were recruited from psychiatric treatment centers and were receiving routine clinical care, including pharmacotherapy and psychotherapy. These treatment conditions may have influenced participants’ emotional functioning and psychological characteristics and should therefore be considered when interpreting the findings. Future studies are encouraged to examine this model in community-based or non-hospitalized BPD samples and to include comparison groups with other psychiatric disorders to improve the generalizability of the findings. Fourth, spiritual intelligence was assessed using a self-report questionnaire. Therefore, participants’ responses may not fully capture the complexity of spirituality and meaning-related processes in individuals with BPD, and the findings should be interpreted with appropriate caution. Fifth, because suicidal ideation was assessed using self-report measures, response bias cannot be completely ruled out.

Finally, the sample consisted predominantly of female participants. Therefore, caution should be exercised when generalizing these findings to male individuals with BPD, and future studies should examine these relationships in more gender-balanced samples. Despite these limitations, the findings provide valuable insights that may inform future research and contribute to the development of psychological interventions for individuals with BPD.

## 5 Conclusion

This study investigated the complex pathways linking emotional intelligence to suicidal ideation among patients with borderline personality disorder (BPD), specifically examining the mediating roles of resilience and spiritual intelligence. The findings support a multi-layered model in which emotional intelligence may serve as a foundational resource that enhances both resilience and spiritual functioning. Importantly, the results suggest that resilience may represent an important mechanism through which emotional competence is associated with adaptive coping, while spiritual intelligence appears to be primarily associated with suicidal ideation through the active construction of personal meaning, rather than through general spiritual awareness alone. By supporting this integrated model, this research contributes significantly to the theoretical understanding of suicidal ideation in BPD. Ultimately, these findings provide empirical evidence for understanding the interconnected roles of emotional, spiritual, and resilient factors in BPD, offering new insights into the psychological mechanisms associated with suicidal ideation in this vulnerable population. These findings may help inform the development of holistic interventions that target these specific psychological dimensions to enhance clinical outcomes.

## Data Availability

The raw data supporting the conclusions of this article will be made available by the authors upon reasonable request, subject to appropriate ethical and confidentiality considerations.
